# Treatment with psychostimulants and atomoxetine in people with psychotic disorders: reassessing the risk of clinical deterioration in a real-world setting

**DOI:** 10.1192/bjp.2023.149

**Published:** 2024-03

**Authors:** Olivier Corbeil, Sébastien Brodeur, Josiane Courteau, Laurent Béchard, Maxime Huot-Lavoie, Elaine Angelopoulos, Samanta Di Stefano, Erica Marrone, Alain Vanasse, Marie-Josée Fleury, Emmanuel Stip, Alain Lesage, Ridha Joober, Marie-France Demers, Marc-André Roy

**Affiliations:** Faculty of Pharmacy, Laval University, Quebec, Canada; Department of Psychiatry and Neurosciences, Laval University, Quebec, Canada; and Institute of Psychiatry, Psychology and Neuroscience, King's College London, UK; PRIMUS Research Group, Research Centre of Sherbrooke University Hospital Center (CRCHUS), Sherbrooke, Canada; PRIMUS Research Group, Research Centre of Sherbrooke University Hospital Center (CRCHUS), Sherbrooke, Canada; and Department of Family Medicine and Urgent Medicine, University of Sherbrooke, Sherbrooke, Canada; Douglas Research Centre, Douglas Mental Health University Institute, Montreal, Canada; and Department of Psychiatry, McGill University, Montreal, Canada; Department de Psychiatry and Addictology, University of Montreal, Montreal, Canada; and Department of Psychiatry and Behavioral Science, College of Medicine and Health Science, United Arab Emirates University, Al Ain, UAE; Department of Psychiatry and Addictology, University of Montreal, Montreal, Canada; and Research Centre, Montreal University Institute of Mental Health, Montreal, Canada; Faculty of Pharmacy, Laval University, Quebec, Canada; and CERVO Research Centre, Quebec, Canada; Department of Psychiatry and Neurosciences, Laval University, Quebec, Canada; and CERVO Research Centre, Quebec, Canada

**Keywords:** Attention-deficit hyperactivity disorders, psychotic disorders/schizophrenia, comorbidity, CNS stimulants, drug or substance interactions and side-effects

## Abstract

**Background:**

Although attention-deficit hyperactivity disorder (ADHD) is often comorbid with schizophrenia spectrum and other psychotic disorders (SZSPD), concerns about an increased risk of psychotic events have limited its treatment with either psychostimulants or atomoxetine.

**Aims:**

To examine whether the risk of hospital admission for psychosis in people with SZSPD was increased during the year following the introduction of such medications compared with the year before.

**Method:**

This was a retrospective cohort study using Quebec (Canada) administrative health registries, including all Quebec residents with a public prescription drug insurance plan and a diagnosis of psychotic disorder, defined by relevant ICD-9 or ICD-10 codes, who initiated either methylphenidate, amphetamines or atomoxetine, between January 2010 and December 2016, in combination with antipsychotic medication. The primary outcome was time to hospital admission for psychosis within 1 year of initiation. State sequence analysis was also used to visualise admission trajectories for psychosis in the year following initiation of these medications, compared with the previous year.

**Results:**

Out of 2219 individuals, 1589 (71.6%) initiated methylphenidate, 339 (15.3%) amphetamines and 291 (13.1%) atomoxetine during the study period. After adjustment, the risk of hospital admission for psychosis was decreased during the 12 months following the introduction of these medications when used in combination with antipsychotics (adjusted HR = 0.36, 95% CI 0.24–0.54; *P* < 0.0001).

**Conclusions:**

These findings suggest that, in a real-world setting, when used concurrently with antipsychotic medication, methylphenidate, amphetamines and atomoxetine may be safer than generally believed in individuals with psychotic disorders.

Attention-deficit hyperactivity disorder (ADHD) is one of the most common psychiatric disorders among children, with an estimated prevalence of 2.2–7.2%, and it persists into adulthood in over 50% of people.^1^ Its presence in adults is associated with poor outcomes, including low academic, occupational, economic and social functioning.^2^ In addition, as many as 80% of adults with ADHD have at least one psychiatric comorbidity, the most common being substance use disorders (SUDs) as well as affective and anxiety disorders.^3^ There is also evidence that children and adolescents with ADHD have a more than fourfold increased risk of schizophrenia spectrum and other psychotic disorders (SZSPD) in adulthood compared with the general population.^4^ Among adolescents and young adults at clinical high risk for SZSPD, a childhood diagnosis of ADHD also appears to predict conversion to SZSPD.^5,6^ Not surprisingly, the prevalence of ADHD in individuals with SZSPD is higher than that observed in the general population, with estimates ranging from 10 to 47%.^7^ Several hypotheses have been raised to explain this association, including a genetic link and common environmental risk factors (e.g. preterm birth, low birth weight, maternal substance use during pregnancy).^8,9^ Furthermore, ADHD is associated with an increased risk of SUD, particularly for cannabis, which is itself a significant risk factor for SZSPD.^10,11^

Available evidence suggests that ADHD is associated with a worse course of SZSPD, including earlier onset, lower academic, social and occupational functioning, as well as a poorer response to treatment.^12^ In addition to its frequent association with SUD, ADHD is linked to poor adherence to pharmacological treatment, which in itself is a leading cause of psychotic relapse and hospital admission in individuals with SZSPD.^13^ Therefore, appropriate treatment of comorbid ADHD with psychostimulants, including amphetamines and methylphenidate, or atomoxetine may promote recovery.^11^ However, their mechanism of action may raise concerns about an increased risk of psychosis in vulnerable individuals.^11^ Indeed, psychostimulants increase the synaptic availability of dopamine and noradrenaline, primarily by inhibiting their presynaptic reuptake, which may appear to be an opposite effect to the postsynaptic blockade of dopamine D_2_ receptors achieved with antipsychotics.^11^ In addition, cases of psychotic events have been linked to the use of psychostimulants in individuals without a history of psychosis, which has led to much reluctance to use them in patients with SZSPD.^14,15^

Recent data by Hollis et al (2019) suggest that, contrary to general beliefs associated with its use, methylphenidate was not linked with an increased risk of psychotic events in adolescents and young adults with a history of psychosis.^16^ This Swedish register-based study included over 23 000 individuals aged 12 to 30 years, of whom 479 reported a history of psychosis. In this subgroup, methylphenidate use did not increase the risk of psychotic events in the 12-week period after its initiation compared with the 12-week period preceding it (incidence rate ratio IRR = 0.95; 95% CI 0.69–1.30). In fact, compared with the same 12-week period preceding its initiation, methylphenidate use was associated with a reduced risk of psychotic events 1 year later (IRR = 0.64; 95% CI 0.45–0.91). Although reassuring, the study has some limitations that must be considered. First, of the 479 individuals with a history of psychosis, only 211 had a formal diagnosis of chronic SZSPD (schizophrenia, persistent delusional disorder or schizoaffective disorder), which is a small sample size to adequately assess the risk of psychotic events associated with methylphenidate use. Second, there was no information regarding other important variables that might also affect this risk, namely, antipsychotic use, medication adherence to both the antipsychotic and the psychostimulant, as well as the doses used. Third, because methylphenidate was the most commonly used ADHD treatment in this study cohort, the risk of psychotic episodes associated with amphetamines, which were previously found to confer a higher risk of psychosis than methylphenidate in a general population sample of more than 220 000 adolescents and young adults treated for ADHD (hazard ratio HR = 1.65; 95% CI 1.31–2.09),^17^ could not be examined. Furthermore, as this is a rare adverse event that can occur after only a few months of treatment, randomised controlled trials are not well suited to adequately assess this risk, hence the importance of observational studies. However, as highlighted in a recent systematic review, existing observational studies have mostly focused on examining the risk of psychosis in unaffected individuals, with Hollis et al being the only study to date to include patients with pre-existing psychosis.^18^

To further evaluate the risk of psychotic relapse associated with ADHD medication, including amphetamines, methylphenidate and atomoxetine, in individuals with SZSPD, we conducted a population-based cohort study. The primary objective of this study was to examine whether the initiation of these treatments in individuals with SZSPD was associated with an increased risk of hospital admission for psychosis in the following 12 months compared with the year prior to initiation.

## Method

### Design and data sources

This retrospective cohort study extracted patient data from the Régie de l'assurance maladie du Québec (RAMQ), which administers universal health insurance for Quebec residents, including physician and hospital coverage. The RAMQ's universal health programme is complemented by a public prescription drug insurance plan (PPDIP) that covers individuals without access to a private drug insurance plan, all last-resort financial assistance recipients and about 90% of individuals aged 65 and over. The RAMQ health databases include patient demographic data, a hospital discharge register (MED-ECHO), physician claims and the PPDIP. Demographic databases contain information about patient age, sex at birth, date of death if deceased and eligibility for PPDIP. MED-ECHO contains primary and secondary diagnoses (ICD-9 before April 2006 and ICD-10 after that date), dates of hospital admission, and medical procedures (e.g. surgical interventions). The physician claims database captures the date and diagnosis (ICD-9 codes used through 2017) of each service provided. The drug database includes information on drugs claimed from community pharmacies by individuals with coverage under the PPDIP. The database does not include in-patient medications. Individual patient records were linked to a unique encrypted identifier to provide demographic, medical and drug information.

The study's design and research approach were guided by the good practices for non-randomised studies of treatment effects using secondary data sources outlined by the International Society for Pharmacoeconomics and Outcomes Research (ISPOR).^19–21^

### Study cohort

Extracted from a large cohort database on severe mental disorders (including schizophrenia, bipolar disorder and other psychotic disorders) spanning the period from 1 January 2002 to 31 December 2017, the study cohort included all out-patients of any age initiating a psychostimulant or atomoxetine between 1 January 2010 and 31 December 2016 with a continuous coverage of PPDIP 1 year before and 1 year after initiation (index date). Only patients with a previous diagnosis (2002 to index date) of SZSPD (ICD-9: schizophrenic disorders (295), paranoid states (297), other non-organic psychoses (298); ICD-10: schizophrenia, schizotypal disorder, persistent delusional disorders, acute and transient psychotic disorders, induced delusional disorder, schizoaffective disorders, other non-organic psychotic disorders, unspecified non-organic psychoses (F20–F29); Supplementary Table 1, available at https://dx.doi.org/10.1192/bjp.2023.149) were included in the study cohort. In order to include only patients who were also treated with an antipsychotic, we excluded patients not claiming any antipsychotic 30 days before and after psychostimulant or atomoxetine initiation.

### Dependent variables

The primary dependent variable is the time until a hospital admission for psychosis (ICD-10: F20–F29; Supplementary Table 1) 1 year after psychostimulant or atomoxetine initiation. The secondary dependent variables are time until hospital admission for mental disorders other than psychosis and time until hospital admission for any mental disorders, either psychotic or non-psychotic disorders (ICD-10: F00–F99; X60-Y36, Y90, Y91, R44–R46; Supplementary Table 2).

### Exposure to psychostimulants and atomoxetine

A patient was considered exposed to the drug from the date(s) a prescription was claimed at a community pharmacy and for the time the drug was provided. If the patient received the first prescription in a hospital, the index date would still be the first out-patient prescription, as no information on drug treatment during hospital admission was available. For each day of the 1-year follow-up period after initiation of ADHD medication, a patient was exposed to three possible categories of drug: methylphenidate, amphetamines and atomoxetine. The index date had a 1-year clearance period without ADHD medication.

### Covariables

The following covariables were assessed as they may potentially influence treatment adherence and hospital admission trajectories: sex at birth (female/male); age at psychostimulant or atomoxetine initiation; low socioeconomic status (defined as being a recipient of social welfare or being ≥65 years of age with pension income supplement) (yes/no); psychosis duration (<2 years; 2–5 years; 5–10 years; ≥10 years); initial prescriber of psychostimulant or atomoxetine (psychiatrist/other clinicians); prescriber of the antipsychotic near the index date (psychiatrist/other clinicians); SUD (yes/no); during the 12-month period prior to psychostimulant or atomoxetine initiation, presence of the followings (yes/no for each): personality disorder diagnosis; use of lithium, divalproex, antidepressants, benzodiazepines or lamotrigine; hospital admission for psychosis, for a mental disorder other than psychosis (e.g. bipolar disorder, depression, anxiety), for any mental disorder (either psychotic or non-psychotic disorder) or for a physical health reason; number of ambulatory visits (including emergency, out-patient and primary care clinics); and comorbidity index (0/≥1). Similar to Charlson's comorbidity index, the comorbidity index selected was proposed by Simard et al and was measured during the year before the index date.^22^ Mental conditions, including alcohol and drug misuse, were excluded from the comorbidity index calculation. The exposure to antipsychotics was assessed using the method described above. Five categories of exposure were possible: oral first-generation antipsychotic (FGA); long-acting injectable (LAI) FGA; oral second-generation antipsychotic (SGA); LAI SGA; and poly-antipsychotic (≥2 antipsychotics).

### Statistical analysis

The psychostimulant/atomoxetine utilisation trajectories (as patterns of use over time) are illustrated using a state sequence analysis (SSA) approach. Antipsychotic utilisation trajectories before and after ADHD medication initiation as well as hospital admission trajectories are also presented using the SSA method. This approach provides a graphical visualisation of longitudinal sequential data, such as care pathways, which allows the identification of specific patterns.^23^

Since the SSA approach is not inferential, we also performed Cox regression modelling with time-dependent psychostimulant/atomoxetine and antipsychotic drug variables, including in the models all of the above covariates identified as potentially confounding using the directed acyclic graph approach (i.e. backdoor criterion; Supplementary Figure 1).^20^ The ‘current use (in the past week)’ approach was used to define exposure to psychostimulants/atomoxetine and antipsychotics. The analyses were carried out using SAS 9.4 and the TraMineR package in R for Windows for the visualisation of the trajectories.^24^

A sensitivity analysis examining the effect of psychostimulant or atomoxetine initiation on an outcome that should not be influenced by such exposure, i.e. hospital admission for non-mental disorders, was conducted to assess whether there was substantial residual confounding.^20^ This analysis was performed by comparing the prevalence of hospital admission for non-mental disorders in the year prior to ADHD medication initiation with the prevalence in the following year using McNemar's non-parametric test for paired data.

A *post hoc* analysis was conducted to examine whether individuals who initiated a psychostimulant or atomoxetine were comparable to the population of individuals with SZSPD without ADHD medication use. Therefore, for each member of the study cohort, we extracted up to four controls matched for sex, year of birth (±3 years), date of psychosis onset (±1 year) and antipsychotic use 30 days before or after the index date.

## Results

**Fig. 1:**
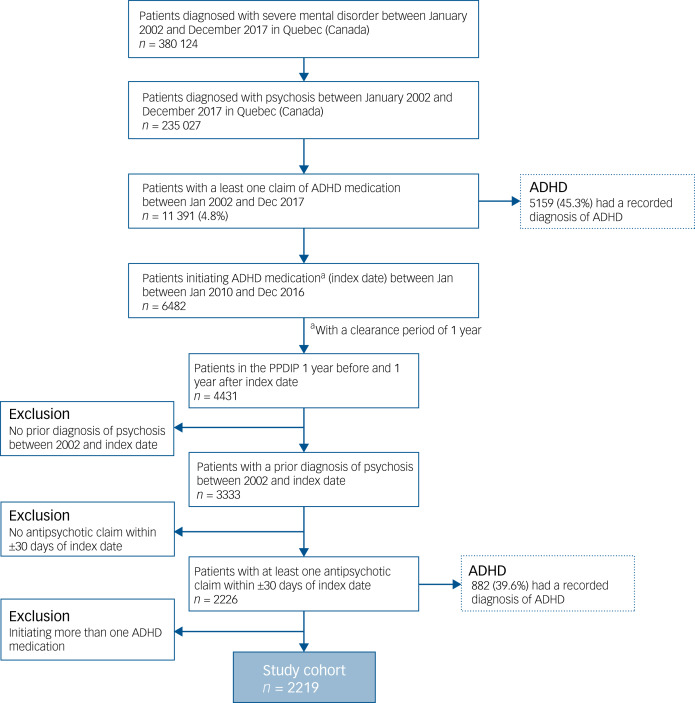
Selection of the study cohort. ADHD, attention-deficit hyperactivity disorder; PPDIP, public prescription drug insurance plan.

There were 235 027 patients diagnosed with SZSPD between January 2002 and December 2017, of whom 11 391 (4.8%) used an ADHD medication during this period (Fig. 1). The final study population consisted of 2219 patients who initiated these medications between 1 January 2010 and 31 December 2016, with a continuous coverage of PPDIP 1 year before and after initiation and at least one antipsychotic claim within 30 days of the index date. There were 1589 (71.6%) patients who initiated methylphenidate, 339 (15.3%) amphetamines and 291 (13.1%) atomoxetine.

### Description of the study cohort

Overall, the study cohort was predominantly male (60.4%), but proportionally more women were prescribed methylphenidate (42.7%) than amphetamines (33.3%) or atomoxetine (30.2%; *P* < 0.0001; Table 1). The mean age of the cohort was 37.0 years, and the methylphenidate group was older on average (mean 38.9 years) than both the amphetamine (33.7 years) and atomoxetine (30.7 years; *P* < 0.0001) groups. There was a greater proportion of participants with low socioeconomic status (*P* = 0.0084), SUDs (*P* = 0.0006), personality disorder (*P* = 0.0302) and prior hospital admission for psychosis (*P* < 0.0001) or any mental disorder, either psychotic or non-psychotic, in the past year (*P* < 0.0001) in the atomoxetine group compared with the other two groups. Duration of psychotic disorder diagnosis averaged 5.6 years and did not differ significantly between the three groups (*P* = 0.5648). Psychiatrists were more often responsible for the initial prescription of both ADHD medication and antipsychotic medication in the atomoxetine group (82.1 and 83.2% respectively) than in the methylphenidate (63.8 and 70.2%) or amphetamine (61.4 and 70.8%) groups.

**Table 1:** Characteristics of the study cohort by type of attention-deficit hyperactivity disorder (ADHD) medication initiated

	Study cohort	Group 1 Methylphenidate	Group 2 Amphetamines	Group 3 Atomoxetine	*P*
Total, *n* (%)	2219	1589 (71.6)	339 (15.3)	291 (13.1)	–
Sex, *n* (%)					<0.0001
Female	879 (39.6)	678 (42.7)	113 (33.3)	88 (30.2)	
Male	1340 (60.4)	911 (57.3)	226 (66.7)	203 (69.8)	
Age, mean (s.d.) (years)	37.0 (15.0)	38.9 (15.9)	33.7 (12.0)	30.7 (10.1)	<0.0001
Low socioeconomic status, *n* (%)	1629 (73.4)	1153 (72.6)	241 (71.1)	235 (80.8)	0.0084
Duration of psychosis diagnosis, mean (s.d.) (years)	5.6 (3.8)	5.7 (3.9)	5.6 (3.9)	5.4 (3.6)	0.5648
Initial prescriber of ADHD medication, *n* (%)					
Psychiatrist	1460 (65.8)	1013 (63.8)	208 (61.4)	239 (82.1)	<0.0001
GP/other MD	759 (34.2)	576 (36.2)	131 (38.6)	52 (17.9)	
Prescriber of antipsychotic, *n* (%)					
Psychiatrist	1597 (72.0)	1115 (70.2)	240 (70.8)	242 (83.2)	<0.0001
GP/other MD	622 (28.0)	474 (29.8)	99 (29.2)	49 (16.8)	
Comorbidity index (≥1), *n* (%)	524 (23.6)	400 (25.2)	61 (18.0)	63 (21.7)	0.0129
Substance-related disorders, *n* (%)	755 (34.0)	506 (31.8)	124 (36.6)	125 (43.0)	0.0006
Personality disorder, *n* (%)	633 (28.5)	437 (27.5)	94 (27.7)	102 (35.0)	0.0302
Lithium use, *n* (%)	269 (12.1)	197 (12.4)	43 (12.7)	29 (10.0)	0.4761
Antidepressant use, *n* (%)	1388 (62.6)	1029 (64.8)	210 (62.0)	149 (51.2)	<0.0001
Benzodiazepine use, *n* (%)	1180 (53.2)	890 (56.0)	162 (47.8)	128 (44.0)	<0.0001
Divalproex use, *n* (%)	337 (15.2)	244 (15.4)	50 (14.8)	43 (14.8)	0.9401
Lamotrigine use, *n* (%)	157 (7.1)	113 (7.1)	29 (8.6)	15 (5.2)	0.2511
Number of ambulatory visits, mean (s.d.)	16.0 (16.5)	16.0 (15.1)	15.9 (22.2)	16.3 (16.0)	0.5007
Prior hospital admission for psychosis, *n* (%)^a^	346 (15.6)	231 (14.5)	45 (13.3)	70 (24.0)	<0.0001
Prior hospital admission for a mental disorder other than psychosis, *n* (%)^a^	579 (26.1)	400 (25.2)	88 (26.0)	91 (31.3)	0.0932
Prior hospital admission for any mental disorder, *n* (%)^a^	822 (37.0)	559 (35.2)	121 (35.7)	142 (48.8)	<0.0001
Prior hospital admission for non-mental disorder, *n* (%)^a^	305 (13.7)	271 (14.7)	55 (10.0)	50 (13.1)	0.0745
Outcomes 1 year after ADHD medication initiation					
Hospital admission for psychosis, *n* (%)	248 (11.2)	168 (10.6)	35 (10.3)	45 (15.5)	0.0446
Hospital admission for a mental disorder other than psychosis, *n* (%)	387 (17.4)	278 (17.5)	58 (17.1)	51 (17.5)	0.9848
Hospital admission for any mental disorder, *n* (%)	564 (25.4)	397 (25.0)	83 (24.5)	84 (28.9)	0.3432
Hospital admission for non-mental disorder, *n* (%)	290 (13.1)	226 (14.2)	39 (11.5)	25 (8.6)	0.0210

ADHD, attention-deficit/hyperactivity disorder; GP, general practitioner; MD, medical doctor; SZSPD, schizophrenia spectrum and other psychotic disorders.

a. ‘Prior' refers to the 12-month period prior to ADHD medication initiation.

### ADHD medication utilisation trajectories

**Fig. 2:**
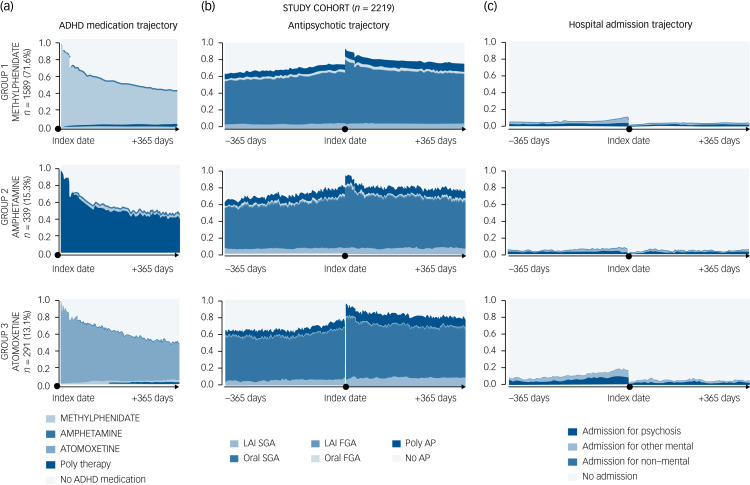
(a) State distribution plots of attention-deficit hyperactivity disorder (ADHD) medication use 1 year after its initiation (index date). (b) Antipsychotic treatment trajectories and (c) hospital admission trajectories 1 year before and 1 year after the index date, stratified by type of medication. FGA, first-generation antipsychotic; LAI, long-acting injectable; SGA, second-generation antipsychotic. The index date has a 1-year clearance period without ADHD medication.

The utilisation of psychostimulants and atomoxetine in the various groups seemed to follow a similar trajectory, i.e. an initial continuous use followed by a decrease in medication adherence over the following year, with approximately 50% of participants not continuously covered by a psychostimulant or atomoxetine 1 year after its initiation (Fig. 2).

### Antipsychotic utilisation trajectories

Although all included participants had to have at least one antipsychotic claim within 30 days before or after ADHD medication initiation, antipsychotic adherence remained high throughout the following year, with an early spike immediately after psychostimulant or atomoxetine initiation (Fig. 2). Oral SGA and poly-antipsychotics were the most commonly used treatments. LAI antipsychotics were used in fewer than 10% of the study cohort.

### Hospital admission trajectories

The SSA visual approach showed an overall reduction in hospital admission rates for psychosis and for mental disorders other than psychosis for all individuals in the year following ADHD medication initiation compared with the previous year (Fig. 2).

### Risk of hospital admission for psychosis

**Fig. 3:**
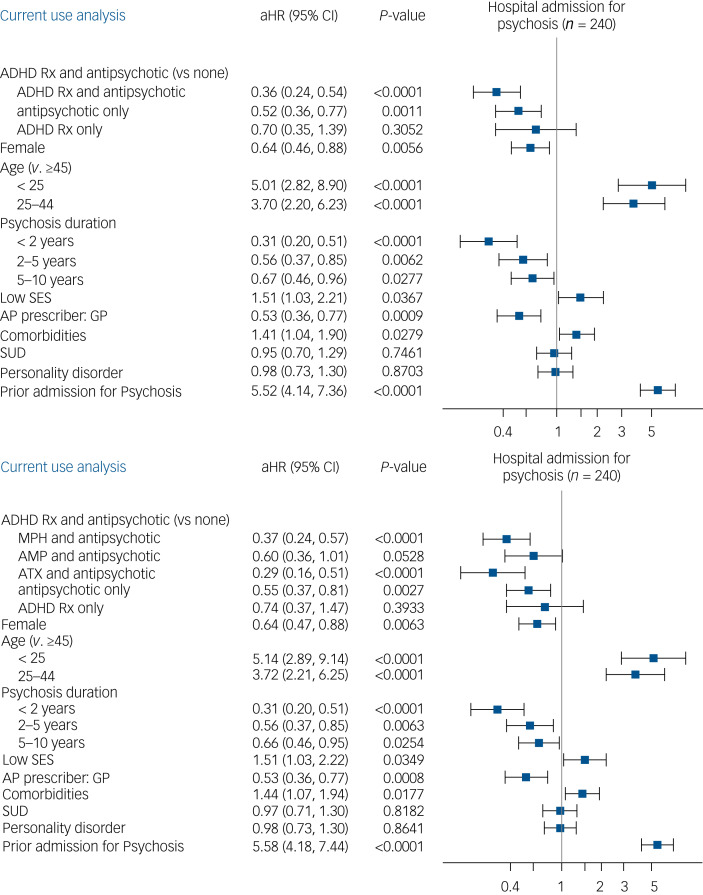
Risk of hospital admission for psychosis in the year after attention-deficit hyperactivity disorder (ADHD) medication initiation. aHR, adjusted hazard ratio; AMP, amphetamine; ATX, atomoxetine; GP, general practitioner; MPH, methylphenidate; Rx, medication; SES, socioeconomic status; SUD, substance use disorder. aHR (95% CI) associated with each covariable.

Cox regression models confirmed the statistical significance of these observations (Fig. 3). Specifically, after adjusting for sex, age, SZSPD duration, low socioeconomic status, antipsychotic prescriber, comorbidity index, history of hospital admission for psychosis, SUDs and personality disorder, there was a lower risk of admission for psychosis among individuals receiving a combination of antipsychotic and psychostimulant or atomoxetine in the year after initiation (adjusted hazard ratio aHR = 0.36, 95% CI 0.24–0.54; *P* < 0.0001), compared with those taking neither treatment at the time of the event (current use approach). The combination of both methylphenidate (aHR = 0.37, 95% CI 0.24–0.57; *P* < 0.0001) or atomoxetine (aHR = 0.29; 95% CI 0.16–0.51; *P* < 0.0001) and antipsychotics was also associated with a decreased risk of hospital admission for psychosis. Although not statistically significant, a similar trend was observed for the use of amphetamines combined with antipsychotics (aHR = 0.60, 95% CI 0.36–1.01; *P* = 0.0528). Comparable results were found when examining the secondary outcomes (i.e. risk of hospital admission for any mental disorder, either psychotic or non-psychotic, or for mental disorders other than psychosis; Supplementary Figs 2 and 3).

### Sensitivity analysis

The prevalence of hospital admission for non-mental disorders in the year prior to psychostimulant or atomoxetine initiation in the study cohort was 13.7%, compared with 13.1% in the subsequent year (Table 1). This difference was not statistically significant (*P* = 0.4572). In comparison, hospital admission for psychosis decreased from 15.6 to 11.2% (*P* < 0.0001), and a similar association was observed for hospital admission for mental disorders other than psychosis (*P* < 0.0001) and for hospital admission for any mental disorder, either psychotic or non-psychotic (*P* < 0.0001).

### *Post hoc* analysis

Overall, 7802 participants were paired to form the comparison group. Participants initiated on an ADHD medication (the study cohort) appeared to have a more complex clinical profile than the comparison group (Supplementary Table 3). SUDs and personality disorders were more prevalent in the study cohort than in the comparison group (34.0% *v.* 18.9% and 28.5% *v.* 12.7% respectively; *P* < 0.0001), as was the use of psychotropic medications other than antipsychotics (i.e. lithium, divalproex, lamotrigine, antidepressants and benzodiazepines). The proportion of hospital admissions for psychosis in the year before the index date was similar in both groups (15.6% *v.* 15.3%; *P* = 0.762). In contrast, the psychostimulants/atomoxetine group had higher rates of admission in the previous year for other mental and non-mental disorders than the comparison group.

## Discussion

### Main findings

In a real-world setting using a large health database, we found that psychostimulants and atomoxetine, when used in combination with antipsychotics, decreased the rates of hospital admission for psychosis and for mental disorders other than psychosis in the year after their initiation compared with the previous year, both graphically and statistically. Moreover, although this finding could have been the result of selection bias, i.e. psychostimulants or atomoxetine being initiated in individuals with less complex clinical profiles than those representing the majority of the SZSPD population, participants who were exposed to these medications appeared instead to have more complex profiles than those who were not exposed to these drugs.

These findings are in line with a previous observational study in which methylphenidate use was not found to increase the risk of psychotic events among adolescents and young adults with a history of psychosis.^16^ In fact, similar to our results, Hollis et al also found evidence supporting a protective association of methylphenidate use on psychosis in their population. Although their sample was limited to methylphenidate and included only 479 individuals, of whom less than half had a formal diagnosis of SZSPD, our findings are supported by a population of 2219 patients representative of real-life settings and extend to both amphetamines and atomoxetine. Furthermore, although a majority of participants received methylphenidate, the results do not suggest that amphetamines are less safe than methylphenidate in terms of hospital admission for psychosis, as observed in a recent study.^17^ These findings suggest that psychostimulant use in certain individuals with SZSPD is safer than is commonly conveyed in the current literature and clinical practice.^11,14,15,18^

Although these findings may seem surprising at first glance, some hypotheses could be raised to explain the results obtained. First, although the participants for whom psychostimulants and atomoxetine were prescribed seemed to have more complex clinical profiles than the comparison group, they nevertheless had greater adherence to their antipsychotic medication, both prior to initiation of ADHD medication and during the year after, than is generally encountered in clinical practice. Although this latter aspect was the result of the inclusion criteria, it is possible that prescribers are more likely to initiate ADHD medication in patients with well-known adherence to their antipsychotic treatment, which would in turn reduce the risk of hospital admission. In parallel, adequate treatment of ADHD could also increase medication adherence in individuals with SZSPD as it improves neurocognitive impairments that negatively affect adherence. However, this study does not allow us to evaluate whether this is the case. Second, it may also be that psychostimulants (and atomoxetine) do not significantly interfere with the pharmacodynamics of co-prescribed antipsychotics. Indeed, although limited, there is evidence that administration of psychostimulants at therapeutic doses does not increase dopamine transmission in the mesolimbic pathway, the region where antipsychotics exert their effects on the positive symptoms associated with psychotic disorders.^11^ Furthermore, psychostimulants may even decrease phasic dopamine release while tuning up tonic liberation, thereby producing attention-enhancing effects without causing psychotic symptoms.^25^

### Strengths and limitations

This is the first North American observational study, and the largest to date, to examine the risk of psychosis associated with ADHD medication use specifically in individuals with SZSPD.^18^ In addition, the use of an extensive health database, which includes the majority of people with psychotic disorders in the province of Quebec, Canada, provides a representative picture of real-world clinical practice. Although randomised clinical trials are widely considered the gold standard for assessing medication efficacy, they may not fully capture the complexity of patients commonly encountered in daily clinical practice, where psychiatric comorbidities such as SUDs and personality disorders are the norm rather than the exception. They may also struggle to assess relatively rare events such as psychotic relapse, particularly in more specific subpopulations such as individuals with psychotic disorders. Consequently, retrospective epidemiological studies based on large secondary databases provide valuable insights that complement the findings of randomised controlled trials.^19^ The database used also provides extensive information on potential confounding variables such as psychosis duration, socioeconomic status, psychiatric and physical comorbidities as well as history of hospital admission, which could be taken into account in the statistical analyses to limit a potential confusion bias. Additionally, the lack of effect of ADHD medication use on an unrelated outcome (i.e. hospital admission for non-mental disorders) provides more confidence in the main results obtained.^20^ The trajectories illustrated using the SSA approach allowed longitudinal measurement of hospital admission patterns, as well as psychostimulant/atomoxetine and antipsychotic utilisation, providing a continuous measure of these complex phenomena compared with traditional dichotomous outcome measures. The comparison of individuals prescribed psychostimulants or atomoxetine with a control group also showed that the results obtained were not entirely due to a possible selection bias. In addition, this study brings forth novel data on the usage of amphetamines and atomoxetine in individuals with SZSPD. The 1-year follow-up period following ADHD medication initiation also supports the persistence over time of the observed results.

The results of this study must be interpreted while considering some limitations. First, information about the specific therapeutic indication for psychostimulant/atomoxetine treatment was not available. Thus, although approximately 40% of all individuals for whom these drugs were prescribed had a formal diagnosis of ADHD, it is possible, but unlikely given that psychostimulants are only indicated for the treatment of ADHD in Canada, that some people received these medications for another indication. However, the purpose of this study was to evaluate the safety of initiating psychostimulants or atomoxetine in people with a diagnosis of SZSPD who were also receiving an antipsychotic. Within this context, although the effectiveness of psychostimulants or atomoxetine use may be strongly contingent on the particular therapeutic purpose for which they are prescribed, their safety concerning the risk of psychotic deterioration should not be substantially, if at all, affected. Second, data on ADHD medication use excluded the period when participants were hospital in-patients, but initiation of these drugs in the context of an acute hospital admission for psychosis is rare in practice, and only 16.3% of the study cohort were in-patients in the month prior to the index date. Third, the study design did not allow us to capture the mean or the maximum dose of psychostimulants or atomoxetine used (Supplementary Table 4). Therefore, it is not possible to conclude whether the results obtained can be explained by the use of lower doses than those commonly used for the treatment of ADHD in the general population. It is also not possible to assess whether the results obtained might be secondary to an increased intensity of clinical follow-up after initiation compared with controls, as this information is not readily available in health registries.

### Implications

For people with SZSPD, attention and executive function problems, whether or not related to ADHD, can significantly interfere with daily functioning and recovery. For these patients who are adequately treated with antipsychotic medications, the use of methylphenidate, amphetamines and atomoxetine in a real-world setting appears to be safer than generally reported in the available literature. These findings support a more proactive and comprehensive management of all comorbidities associated with SZSPD.

## Supporting information

Corbeil et al. supplementary material Corbeil et al. supplementary material

## Data Availability

The data are not publicly available because of privacy or ethical restrictions.
