# Supplementary material for: Treatment with psychostimulants and atomoxetine in people with psychotic disorders: reassessing the risk of clinical deterioration in a real-world setting
Source: Br J Psychiatry. 2024 Mar;224(3):98–105. doi: 10.1192/bjp.2023.149 (PMC10884826; doi:10.1192/bjp.2023.149)
Supplement: Corbeil et al. supplementary material [file S0007125023001496sup001.docx]

# Supplementary Figure 1. Directed acyclic graphs for the main analysis.


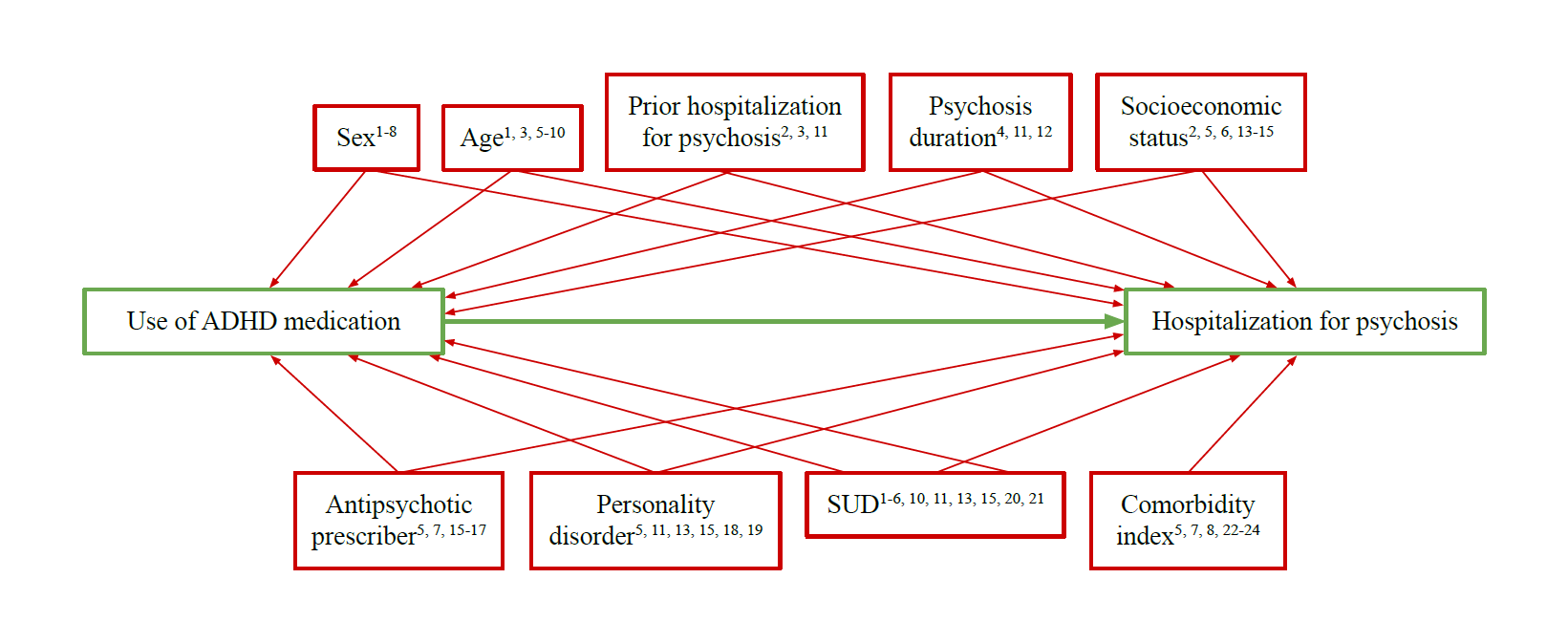


*SUD, substance use disorder.*

**References**

**1.** Rubio JM, Schoretsanitis G, John M, Tiihonen J, Taipale H, Guinart D, et al. Psychosis relapse during treatment with long-acting injectable antipsychotics in individuals with schizophrenia-spectrum disorders: an individual participant data meta-analysis. *Lancet Psychiatry* 2020; **7**(9): 749-61.

**2.** Bowtell M, Ratheesh A, McGorry P, Killackey E, O'Donoghue B. Clinical and demographic predictors of continuing remission or relapse following discontinuation of antipsychotic medication after a first episode of psychosis. A systematic review. *Schizophr Res* 2018; **197**: 9-18.

**3.** Olivares JM, Sermon J, Hemels M, Schreiner A. Definitions and drivers of relapse in patients with schizophrenia: a systematic literature review. *Ann Gen Psychiatry* 2013; **12**(1): 32.

**4.** Schoeler T, Petros N, Di Forti M, Pingault JB, Klamerus E, Foglia E, et al. Association between continued cannabis use and risk of relapse in first-episode psychosis: a quasi-experimental investigation within an observational study. *JAMA Psychiatry* 2016; **73**(11): 1173-9.

**5.** Martinez-Raga J, Ferreros A, Knecht C, de Alvaro R, Carabal E. Attention-deficit hyperactivity disorder medication use: factors involved in prescribing, safety aspects and outcomes. *Ther Adv Drug Saf* 2017; **8**(3): 87-99.

**6.** Carolan D. ADHD stimulant medication misuse and considerations for current prescribing practice: a literature review. *Ir J Med Sci* 2022; **191**(1): 313-20.

**7.** Sibley MH. Why are stimulant medication prescriptions rising globally? *Lancet Psychiatry* 2018; **5**(10): 774-6.

**8.** Brumbaugh S, Tuan WJ, Scott A, Latronica JR, Bone C. Trends in characteristics of the recipients of new prescription stimulants between years 2010 and 2020 in the United States: an observational cohort study. *EClinicalMedicine* 2022; **50**: 101524.

**9.** Hui CL, Tang JY, Leung CM, Wong GH, Chang WC, Chan SK, et al. A 3-year retrospective cohort study of predictors of relapse in first-episode psychosis in Hong Kong. *Aust N Z J Psychiatry* 2013; **47**(8): 746-53.

**10.** Mattingly GW, Wilson J, Ugarte L, Glaser P. Individualization of attention-deficit/hyperactivity disorder treatment: pharmacotherapy considerations by age and co-occurring conditions. *CNS Spectr* 2021; **26**(3): 202-21.

**11.** Levy E, Traicu A, Iyer S, Malla A, Joober R. Psychotic disorders comorbid with attention-deficit hyperactivity disorder: an important knowledge gap. *Can J Psychiatry* 2015; **60**(3 Suppl 2): S48-52.

**12.** Pothimas N, Bhatarasakoon P, Chanprasit C, Kitsumban V. A cross-sectional study of factors predicting relapse in people with schizophrenia. *Pacific Rim Int J Nurs Res* 2020; **24**(4): 448-59.

**13.** Corbeil O, Essiambre AM, Béchard L, Roy AA, Huot-Lavoie M, Brodeur S, et al. Real-life effectiveness of transitioning from paliperidone palmitate 1-monthly to paliperidone palmitate 3-monthly long-acting injectable formulation. *Ther Adv Psychopharmacol* 2022; **12**: 20451253221136021.

**14.** Almuqrin A, Georgiades A, Mouhitzadeh K, Rubinic P, Mechelli A, Tognin S. The association between psychosocial stress, interpersonal sensitivity, social withdrawal and psychosis relapse: a systematic review. *Schizophrenia (Heidelb)* 2023; **9**(1): 22.

**15.** Puntis S, Oke J, Lennox B. Discharge pathways and relapse following treatment from early intervention in psychosis services. *BJPsych Open* 2018; **4**(5): 368-74.

**16.** Fusar-Poli P, McGorry PD, Kane JM. Improving outcomes of first-episode psychosis: an overview. *World Psychiatry* 2017; **16**(3): 251-65.

**17.** Stockl KM, Hughes TE, Jarrar MA, Secnik K, Perwien AR. Physician perceptions of the use of medications for attention deficit hyperactivity disorder. *J Manag Care Pharm* 2003; **9**(5): 416-23.

**18.** Strålin P, Hetta J. First episode psychosis: register-based study of comorbid psychiatric disorders and medications before and after. *Eur Arch Psychiatry Clin Neurosci* 2021; **271**(2): 303-13.

**19.** Bahorik AL, Eack SM. Examining the course and outcome of individuals diagnosed with schizophrenia and comorbid borderline personality disorder. *Schizophr Res* 2010; **124**(1-3): 29-35.

**20.** Harstad E, Levy S. Attention-deficit/hyperactivity disorder and substance abuse. *Pediatrics* 2014; **134**(1): e293-301.

**21.** Brikell I, Wimberley T, Albiñana C, Pedersen EM, Vilhjálmsson BJ, Agerbo E, et al. Genetic, clinical, and sociodemographic factors associated with stimulant treatment outcomes in ADHD. *Am J Psychiatry* 2021; **178**(9): 854-64.

**22.** Filipcic I, Simunovic Filipcic I, Ivezic E, Matic K, Tunjic Vukadinovic N, Vuk Pisk S, et al. Chronic physical illnesses in patients with schizophrenia spectrum disorders are independently associated with higher rates of psychiatric rehospitalization; a cross-sectional study in Croatia. *Eur Psychiatry* 2017; **43**: 73-80.

**23.** Šprah L, Dernovšek MZ, Wahlbeck K, Haaramo P. Psychiatric readmissions and their association with physical comorbidity: a systematic literature review. *BMC Psychiatry* 2017; **17**(1): 2.

**24.** Köhler O, Petersen L, Benros ME, Mors O, Gasse C. Concomitant NSAID use during antipsychotic treatment and risk of 2-year relapse - a population-based study of 16,253 incident patients with schizophrenia. *Expert Opin Pharmacother* 2016; **17**(8): 1055-62.

# Supplementary Figure 2. Risk of hospitalization for mental disorders (including psychosis) in the year after ADHD medication initiation.

*
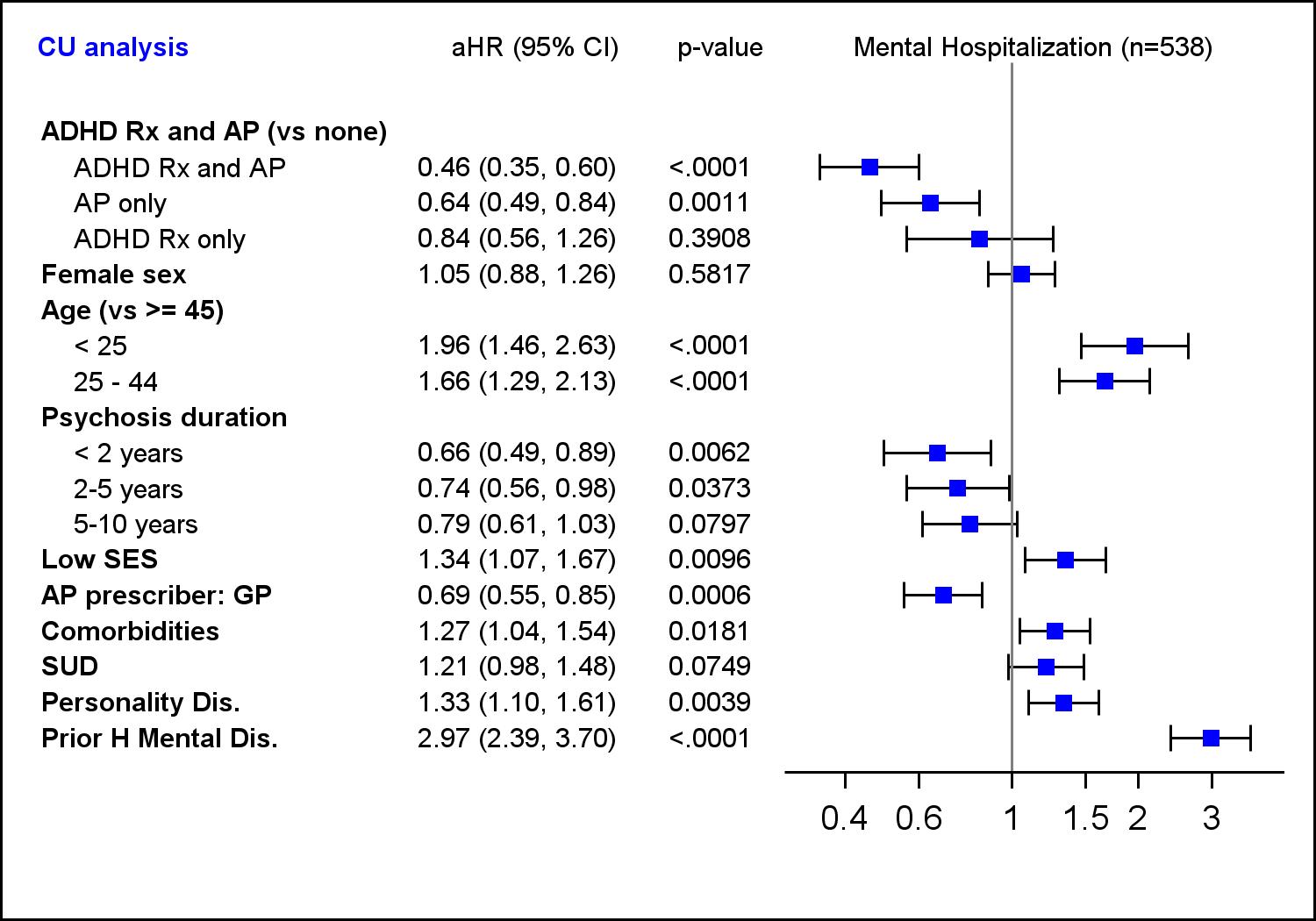

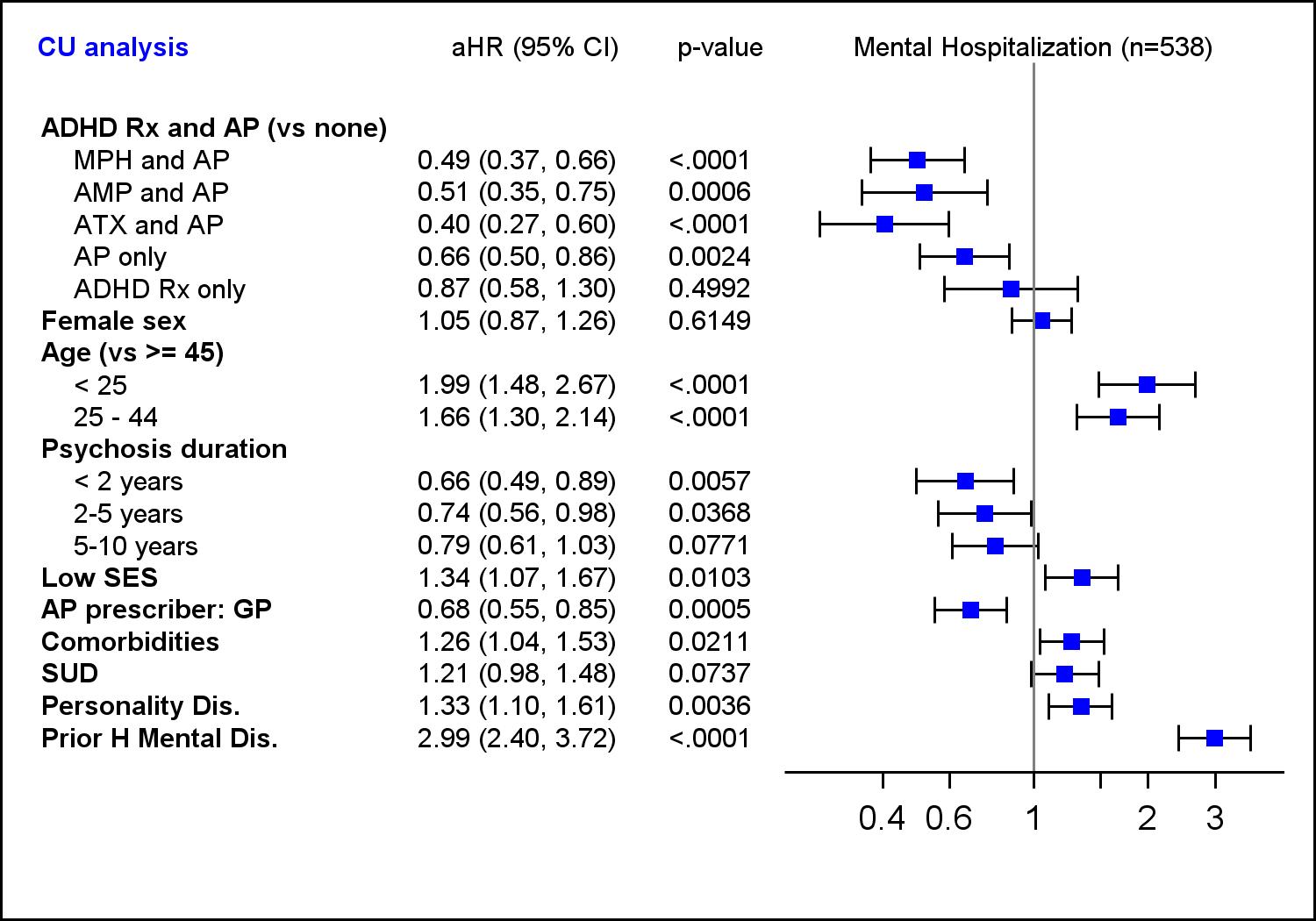
*

*ADHD, attention-deficit/hyperactivity disorder; aHR, adjusted hazard ratio; AMP, amphetamine; AP, antipsychotic; ATX, atomoxetine; CI, confidence interval; CU, current use; GP, general practitioner; H, hospitalization; MPH, methylphenidate; Rx, medication; SES, socioeconomic status; SUD, substance use disorder.*

*aHR (95% CI) associated with each covariable.*

# Supplementary Figure 3. Risk of hospitalization for mental disorders other than psychosis in the year after ADHD medication initiation.

*
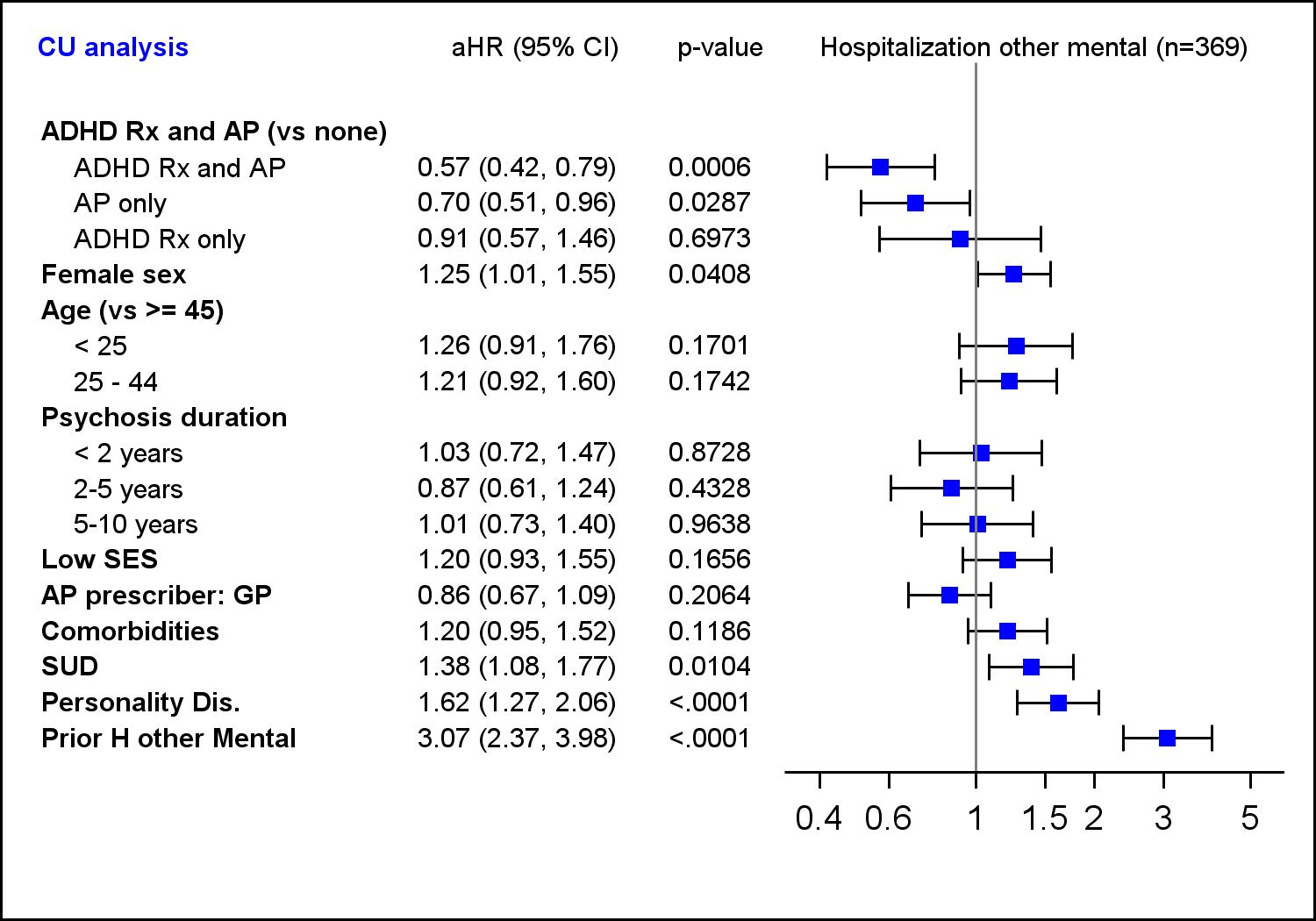
*

*
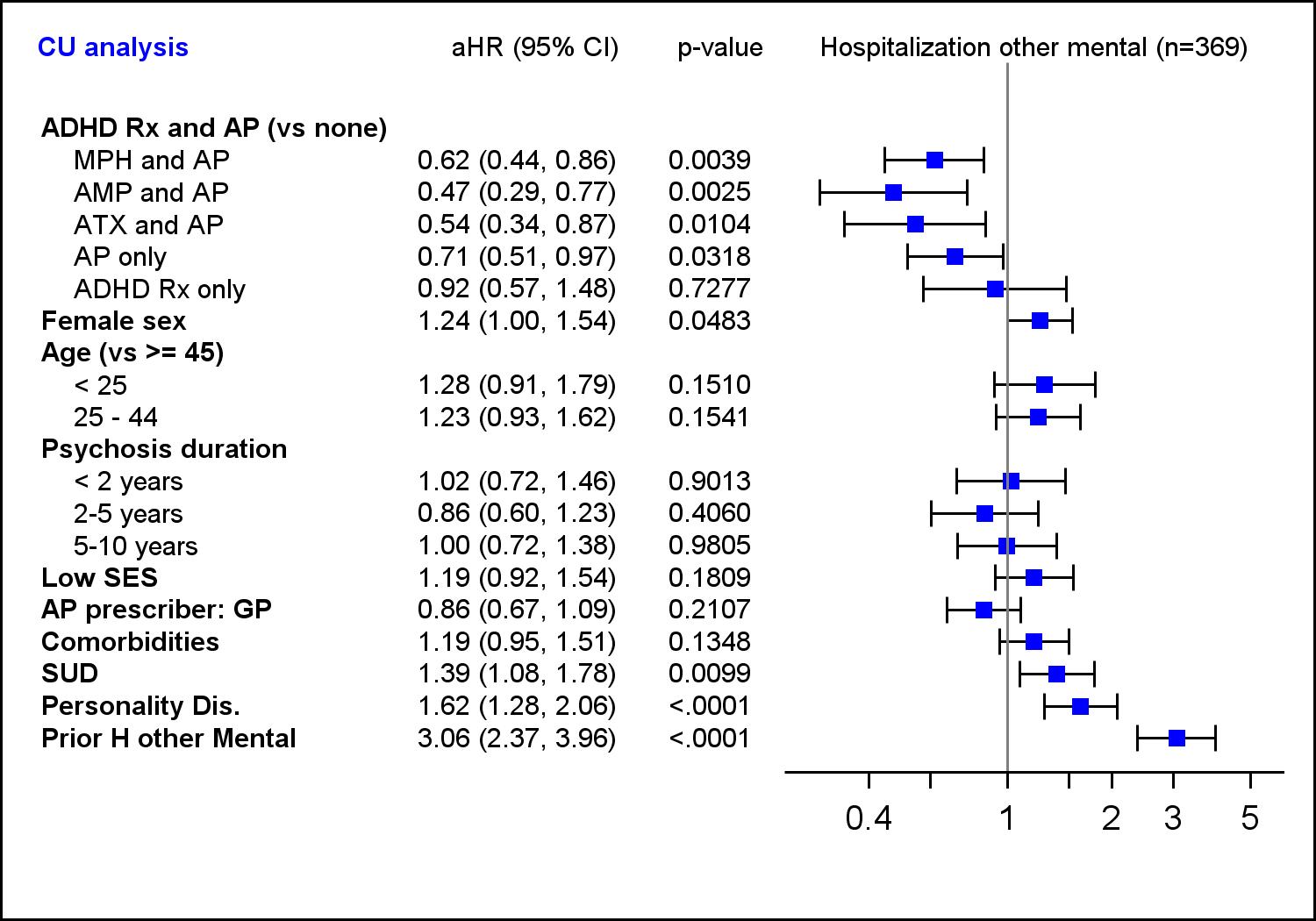
*

*ADHD, attention-deficit/hyperactivity disorder; aHR, adjusted hazard ratio; AMP, amphetamine; AP, antipsychotic; ATX, atomoxetine; CI, confidence interval; CU, current use; GP, general practitioner; H, hospitalization; MPH, methylphenidate; Rx, medication; SES, socioeconomic status; SUD, substance use disorder*

*aHR (95% CI) associated with each covariable.*

# **Supplementary Table 1.** List and description of ICD-9 and ICD-10 for psychosis.

| **ICD-9** | **Description** | **ICD-10** | **Description** |
| --- | --- | --- | --- |
| **295** | **Schizophrenic disorders** | **F20** | **Schizophrenia** |
| 295.0 | Simple type | F20.0 | Paranoid schizophrenia |
| 295.1 | Hebephrenic type | F20.1 | Hebephrenic schizophrenia |
| 295.2 | Catatonic type | F20.2 | Catatonic schizophrenia |
| 295.3 | Paranoid type | F20.3 | Undifferentiated schizophrenia |
| 295.4 | Acute schizophrenic episode | F20.4 | Post-schizophrenic depression |
| 295.5 | Latent schizophrenia | F20.5 | Residual schizophrenia |
| 295.6 | Residual schizophrenia | F20.6 | Simple schizophrenia |
| 295.7 | Schizo-affective type | F20.8 | Other |
| 295.8 | Other | F20.9 | Unspecified |
| 295.9 | Unspecified | **F21** | **Schizotypal disorder** |
| **297** | **Paranoid states** | **F22** | **Persistent delusional disorders** |
| 297.0 | Paranoid state, simple | F22.0 | Delusional disorder |
| 297.1 | Paranoia | F22.8 | Other |
| 297.2 | Paraphrenia | F22.9 | Unspecified |
| 297.3 | Induced psychosis | **F23** | **Acute and transient psychotic disorders** |
| 297.8 | Other | F23.0 | Acute polymorphic psychotic disorder without symptoms of schizophrenia |
| 297.9 | Unspecified | F23.1 | Acute polymorphic psychotic disorder with symptoms of schizophrenia |
| **298** | **Other nonorganic psychoses** | F23.2 | Acute schizophrenia-like psychotic disorder |
| 298.0 | Depressive type | F23.3 | Other acute predominantly delusional psychotic disorders |
| 298.1 | Excitative type | F23.8 | Other |
| 298.2 | Reactive confusion | F23.9 | Unspecified |
| 298.3 | Acute paranoid reaction | **F24** | **Induced delusional disorder** |
| 298.4 | Psychogenic paranoid psychosis | **F25** | **Schizoaffective disorders** |
| 298.8 | Other and unspecified reactive psychosis | F25.0 | Manic type |
| 298.9 | Unspecified psychosis | F25.1 | Depressive type |
|  |  | F25.2 | Mixed type |
|  |  | F25.8 | Other |
|  |  | F25.9 | Unspecified |
|  |  | **F28** | **Other nonorganic psychotic disorders** |
|  |  | **F29** | **Unspecified nonorganic psychosis** |

# Supplementary Table 2. List and description of ICD-10 for mental disorders.

| **ICD-10** | **Description** |
| --- | --- |
| **F00-F99** | **Mental and behavioural disorders** |
| F00-F09 | Organic, including symptomatic, mental disorders |
| F10-F19 | Mental and behavioural disorders due to psychoactive substance use |
| F20-F29 | Schizophrenia, schizotypal and delusional disorders |
| F30-F39 | Mood [affective] disorders |
| F40-F49 | Neurotic, stress-related and somatoform disorders |
| F50-F59 | Behavioural syndromes associated with physiological disturbances and physical factors |
| F60-F69 | Disorders of adult personality and behaviour |
| F70-F79 | Mental retardation |
| F80-F89 | Disorders of psychological development |
| F90-F98 | Behavioural and emotional disorders with onset usually occurring in childhood and adolescence |
| F99 | Unspecified mental disorder |
| **R44-R46** | **Symptoms and signs involving cognition, perception, emotional state and behaviour** |
| R44 | Other symptoms and signs involving general sensations and perceptions - hallucinations |
| R45 | Symptoms and signs involving emotional state |
| R46 | Symptoms and signs involving appearance and behaviour |
| **X60-Y91** |  |
| X60-X84 | Intentional self-harm |
| X85-Y09 | Assault |
| Y10-Y34 | Event of undetermined intent |
| Y35-Y36 | Legal intervention and operations of war |
| Y90 | Evidence of alcohol involvement determined by blood alcohol level |
| Y91 | Evidence of alcohol involvement determined by level of intoxication |

# Supplementary Table 3. Comparison between the study cohort and the comparison group.

| Baseline characteristics | COHORT | COMPARISON GROUP^a^ | p-value |
| --- | --- | --- | --- |
| Total, n | 2219 | 7802 | - |
| Sex, n (%)  Female  Male | 879 (39.6)  1340 (60.4) | 3056 (39.2)  4746 (60.8) | .7061 |
| Age, mean ± SD | 37.0 (15.0) | 38.3 (14.5) | .0003 |
| Low socioeconomic status, n (%) | 1629 (73.4) | 5998 (76.9) | .0007 |
| Duration of SZSPD diagnosis, mean ± SD | 5.6 (3.8) | 6.0 (3.9) | .0001 |
| Initial prescriber of ADHD medication, n (%)  Psychiatrist  General practitioner  Other MD | 1460 (65.8)  711 (32.0)  48 (2.2) | - | - |
| Prescriber of AP, n (%)  Psychiatrist  General practitioner  Other MD | 1597 (72.0)  599 (27.0)  23 (1.0) | 5378 (68.9)  2338 (30.0)  86 (1.1) | .0227 |
| Comorbidity index (≥ 1), n (%) | 524 (23.6) | 1364 (17.5) | <.0001 |
| Substance-related disorders, n (%) | 755 (34.0) | 1472 (18.9) | <.0001 |
| Personality disorder, n (%) | 633 (28.5) | 991 (12.7) | <.0001 |
| Lithium use, n (%) | 269 (12.1) | 730 (9.4) | .0001 |
| Antidepressant use, n (%) | 1388 (62.6) | 3316 (42.5) | <.0001 |
| Benzodiazepine use, n (%) | 1180 (53.2) | 3099 (39.7) | <.0001 |
| Divalproex use, n (%) | 337 (15.2) | 1047 (13.4) | .0332 |
| Lamotrigine use, n (%) | 157 (7.1) | 252 (3.2) | <.0001 |
| Number of ambulatory visits, mean ± SD | 16.0 (16.5) | 12.2 (14.5) | <.0001 |
| Prior hospitalization for psychosis, n (%) | 346 (15.6) | 1196 (15.3) | .7617 |
| Prior hospitalization for other mental dis., n (%) | 579 (26.1) | 873 (11.2) | <.0001 |
| Prior hospitalization for mental dis., n (%) | 822 (37.0) | 1912 (24.5) | <.0001 |
| Prior hospitalization for non-mental dis., n (%) | 305 (13.7) | 745 (9.6) | <.0001 |

*ADHD, attention-deficit/hyperactivity disorder; AP, antipsychotic; MD, medical doctor; SD, standard deviation; SZSPD, schizophrenia spectrum psychotic disorder.*

*^a^Patients in the comparison group were matched for sex, birth year (±3 years), date of first psychosis (±1 year), and use of AP 30 days before or after the index date.*

# Supplementary Table 4. Initial dosage of ADHD medication.

| Initial ADHD medication | n | Average (SD) | Median (IQR) |
| --- | --- | --- | --- |
| Methylphenidate | 1589 | 21 mg (14 mg) | 18 mg (10 – 27 mg) |
| Amphetamines |  |  |  |
| Dexamphetamine | 78 | 12 mg (8 mg) | 10 mg (10 – 15 mg) |
| Amphetamine | 64 | 18 mg (14 mg) | 80 mg (40 – 120 mg) |
| Lisdexamfetamine | 197 | 26 mg (9 mg) | 30 mg (20 – 30 mg) |
| Atomoxetine | 291 | 38 mg (21 mg) | 40 mg (25 – 40 mg) |

*ADHD, attention-deficit/hyperactivity disorder; IQR, interquartile range; mg, milligram; SD, standard deviation.*
